# Association of Aneurysm Tissue Neutrophil Mediator Levels with Intraluminal Thrombus Thickness in Patients with Abdominal Aortic Aneurysm

**DOI:** 10.3390/biom12020254

**Published:** 2022-02-04

**Authors:** Aldona Siennicka, Monika Adamowicz, Natalie Grzesch, Magdalena Kłysz, Jarosław Woźniak, Miłosław Cnotliwy, Katarzyna Galant, Maria Jastrzębska

**Affiliations:** 1 Department of Laboratory Diagnostics, Pomeranian Medical University, Powstańców Wlkp. 72, 70-111 Szczecin, Poland; aldona.siennicka@pum.edu.pl (A.S.); monada@pum.edu.pl (M.A.); natalia.grzes@pum.edu.pl (N.G.); maria.jastrzebska@pum.edu.pl (M.J.); 2 Institute of Mathematics, University of Szczecin, Wielkopolska 15, 70-451 Szczecin, Poland; jaroslaw.wozniak@usz.edu.pl; 3 Department of Vascular Surgery and Angiology, Pomeranian Medical University, Powstańców Wlkp. 72, 70-111 Szczecin, Poland; mcnotliwy@wp.pl; 4 Department of Laboratory Medicine, Chair of Microbiology, Immunological Diagnostics and Laboratory Medicine, Pomeranian Medical University, Powstańców Wlkp. 72, 70-111 Szczecin, Poland; kasieg231@wp.pl

**Keywords:** abdominal aortic aneurysm, intraluminal thrombus, neutrophil mediators

## Abstract

An intraluminal thrombus (ILT), which accumulates large numbers of neutrophils, plays a key role in abdominal aortic aneurysm (AAA) pathogenesis. This study aimed to compare levels of selected neutrophil inflammatory mediators in thick and thin ILT, plus adjacent AAA walls, to determine whether levels depend on ILT thickness. Neutrophil mediator levels were analysed by enzyme-linked immunosorbent assays in thick and thin segments of ILT, plus adjacent aneurysm wall sections, taken from one aneurysm sac each from 36 AAA patients. In aneurysmal walls covered by thick ILT, neutrophil elastase and TNF-a levels were significantly higher, as were concentrations of IL-6, in thick ILT compared to thin layers. Positive correlations of NGAL, MPO, and neutrophil elastase were observed between thick ILT and the adjacent wall and thin ILT and the adjacent wall, suggesting that these mediators probably infiltrate thick AAA compartments as well as thin. These observations might support the idea that neutrophil mediators and inflammatory cytokines differentially accumulate in AAA tissues according to ILT thickness. The increased levels of neutrophil mediators within thicker AAA segments might suggest the existence of an intensified proinflammatory state that in turn presumably might preferentially weaken the AAA wall at that region.

## 1. Introduction

The development of an AAA results from pathological changes within the aortic wall, leading to the destruction of the vascular media, including smooth muscle cell (SMC) apoptosis, elastin fragmentation, and medial neoangiogenesis [1,2]. Histological examination of an AAA wall reveals transmural inflammatory cell infiltrates in the adventitia and tunica media. These pathological changes are also associated with AAA expansion and rupture [3]. Most AAAs are characterised by having an intraluminal thrombus (ILT) [4,5,6], the formation of which depends on local haemodynamics and morphological features of the aneurysm [5]. Recently, it has been demonstrated that ILT within AAAs were independently associated with faster aneurysm growth [7]. Increasing evidence suggests that an ILT can play important biological and mechanical roles in an AAA’s pathogenesis, including participating in potentially catastrophic rupture [6]. However, the influence of an ILT on aneurysm rupture risk remains controversial, presumably being due to competing mechanical and biochemical effects. Several studies have reported that an ILT generates an inflammatory environment and may accumulate large numbers of polymorphonuclear neutrophils (PMNs), cytokines, and proteases, presumably decreasing AAA wall strength [8,9]. In contrast, an ILT has also been reported to act as a mechanical buffer by reducing aortic wall stress [10].

An AAA is usually asymmetric, and an ILT is often circumferential with the thickest part on the AAA’s ventral side [11,12,13,14]. The thickness of a thrombus and/or the aortic wall may differ between different patients or along each aneurysm [5,15,16]. A fully formed thrombus has a multilayered structure, but it should be noted that not all ILT contain all layers. In the sac of one AAA, there can be thin and thick ILT regions [6,17]. A thin region of an ILT (<10 mm across) may consist of a luminal-type layer in direct contact with the aneurysm wall, while a thicker region of a thrombus (>25 mm across) may have a combination of distinct layers, described as luminal (nearest the blood), medial, and abluminal [18]. It has been suggested that aneurysmal wall remodelling and inflammation generation in aneurysmal tissue might depend on, or be influenced by, the particular structure of an ILT [19]. It has been shown that the luminal layer of an AAA is abundant in PMNs as well as platelets and red blood cells [20]. Thin thrombus allows blood cells and plasma factors to penetrate from the lumen of the aneurysm to the inside of the thrombus and to the aneurysm wall adjacent to it. Wall-derived factors, accumulated directly by the thrombus or adsorbed from the serum, can induce proteolysis and clotting processes, contributing to increased activation of these factors. Increased inflammation in the luminal layer of a thrombus can be part of the chain of responses leading to the destruction of the aneurysm wall and, as a result, its rupture. It has been suggested that the thicker the thrombus, the greater the barrier against migration of pro-inflammatory cells from the blood [4,5]. 

Neutrophils are highly active cells that produce large amounts of proteolytic enzymes, including myeloperoxidase (MPO), neutrophil elastase, neutrophil gelatinase-associated lipocalin (NGAL) or interleukin-6 (IL-6), and tumour necrosis factor (TNF-a) [21]. The presence of PMNs within the ILT and AAA wall may be connected with the main pathological mechanisms of AAA: medial destruction, oxidative stress, and inflammatory processes [21,22]. PMN mediators, NGAL, and neutrophil elastase can upregulate matrix metalloproteinase-9 (MMP-9) and prevent its inactivation [16,19,23]. A cohort study showed strong association between elevated neutrophil counts and AAAs [24]. Myeloperoxidase is most abundantly expressed in neutrophil granulocytes. It is also found in monocytes and macrophages, although in much smaller amounts [25]. PMNs from AAA patients displayed higher H(2)O(2) and myeloperoxidase levels than controls, suggesting that myeloperoxidase has potent oxidant enzymatic activity [26]. The proinflammatory cytokine TNF-a may be a key mediator in aneurysm development as it activates cytokines and MMPs and promotes neutrophil activation [27,28]. Further, the multifunctional cytokine IL-6 contributes to acute and chronic inflammatory processes, adding to the degradation of the aortic wall structure [29,30,31,32]. Several factors in ILT and AAA walls originate from activated neutrophils and can also be detected at increased concentrations in the peripheral blood of patients with an AAA [33,34,35]. It has been reported that by limiting aortic wall neutrophil infiltration, one can inhibit experimental AAA formation [20]. Furthermore, preoperative doxycycline therapy in humans has previously been reported to improve the AAA proteolytic balance [36]. These data also highlight the value of analysing PMN mediators as potential AAA biomarkers.

The former studies focused mainly on the characterisation and localisation of neutrophil markers or mediators in the aneurysmal wall or ILT. Fragments of aneurysm wall covered with ILT or AAA wall segments directly adjacent to flowing blood were examined. Most findings were based on a single sample from each AAA and only at the area of maximal ILT deposition [8,18,19,37,38]. Only a few reports have evaluated an ILT in terms of variation in parameter levels resulting from wall thickness in a sac of the same aneurysm [16,17,39]. As it is strongly suggested that ILT thickness is related to the risk of AAA rupture and may be a prognostic factor, we decided to investigate regional differences in neutrophil mediator levels in thrombus segments with different thicknesses and adjacent segments of the aneurysm walls. In the present study, we hypothesised that PMN mediators and inflammatory cytokines might differentially accumulate in AAA tissues according to ILT thickness. Therefore, we compared patient AAA tissue samples to determine whether ILT thickness influenced levels of inflammatory mediators in human ILT and AAA wall tissues. The aims of the study were: (i) the measurement of neutrophil mediators and inflammatory cytokines, NGAL, MPO, neutrophil elastase, TNF-a, and IL-6 in tissues taken from thick and thin segments of intraluminal thrombus and adjacent AAA walls; (ii) the determination of corresponding plasma/serum levels in the patients and in a control group; and (iii) the evaluation of possible associations between measured parameters. Further understanding of the mechanisms of development and progression of AAAs may provide an important step in explaining the pathophysiology and role of mediators and help in developing and improving biomarkers and new therapeutic strategies for patients with AAA.

## 2. Materials and Methods

### 2.1. Patients

The study group consisted of 36 patients with diagnosed AAA (27 men and 9 women; age range 56 to 84 years old) examined at the Department of Vascular and General Surgery and Angiology at the Pomeranian Medical University in Szczecin, Poland. Inclusion criteria included (revealed by pre-surgery CT scan) the presence of ILT with thin and thick regions, as well as an eccentric blood-flow lumen in one aneurysm sac. Full-thickness AAA samples (ILT with a minimum width of 25 mm at the largest width and maximum width of 10 mm at the thinnest width and adjacent walls separately) were collected during aneurysm repair procedures from patients with AAA with significant ILT. Such inclusion criteria differentiate a thin ILT (up to 10 mm), which permits penetration of blood components to the aneurysm wall from thick ILT (dimensions following [17]). Mean intraluminal thrombus thickness was calculated as the difference between maximal aortic diameter and the lumen’s diameter (these were the circulating aortic channels, measured at the level of maximal dilatation on CT scan) [37]. The mean maximal AAA diameter was 59 ± 12 mm (range: 42 to 100 mm). The mean ILT thickness was 32 ± 10 mm (range: 8 to 56 mm). The control group consisted of 30 patients (21 men and 9 women; age range 51 to 80 years old) with a normal infrarenal aortic diameter (less than 30 mm) treated in the vascular outpatient clinic. Co-existing renal dysfunction, liver dysfunction, and haematological disorders were exclusion criteria for both groups. 

### 2.2. Blood Sampling

Peripheral venous blood samples were taken from controls and each AAA patient preoperatively and before any blood product transfusion or heparin administration. Blood samples were collected in tubes containing ethylenediaminetetraacetic acid (EDTA) to analyse NGAL and tubes with clot activator for analysis of neutrophil elastase, MPO, IL-6, and TNF-a. Plasma and serum were prepared from the blood by centrifugation, frozen in aliquots, and stored at −80 °C until detection. 

### 2.3. Tissue Samples

**Figure 1:**
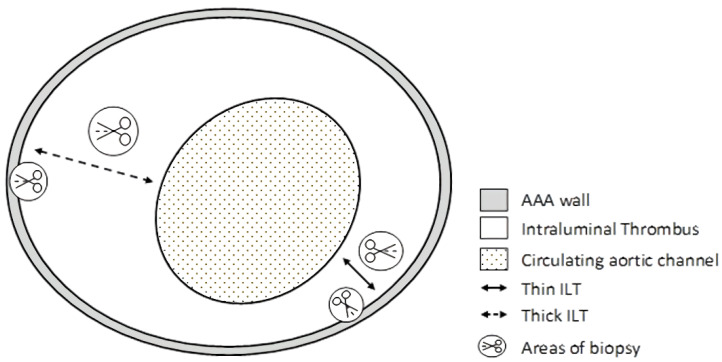
Transverse view of representative abdominal aortic aneurysm, demonstrating the sites of sampling. Arrows point out areas of biopsy: a thick section with a thickness ≥25 mm; a thin section with a thickness of ≤10 mm plus each of two sections of the wall: one section adjacent to the thick part of the ILT and one section adjacent to the thin part of the ILT.

During the elective open repair of AAA, four tissue samples were taken from regions of AAA with high and low ILT deposition from each patient, each 5 × 5 mm. Tissue samples included two sections from the ITL: a thin one and a thick one. Additionally, two sections of the aneurysm wall were collected: one adjacent to ILT’s thin section and one adjacent to the ILT’s thick section. All four tissue samples were harvested from one aneurysm sac (Figure 1). Tissue samples were always obtained from the middle third of the longitudinal dimension of the ILT, close to the position of the AAA sac’s maximum dilatation at the same level. The thickness of the thrombus from each site (before the excision of specimens) was measured using a laser micrometre. Retrieved samples were placed in ice and transported to the laboratory immediately. The aortic tissue was divided into sections, agitated in phosphate-buffered saline to remove blood, weighed, and stored at −80 °C until analysis.

### 2.4. Tissue Homogenisation for Protein Analysis

The aneurysm samples (0.15 to 0.40 g wet mass) were pulverised in liquid N2 and homogenised in lysis buffer containing 150 mM NaCl, 10 mmol/L Tris-HCl (pH 7.4), and 0.25% Triton X-100 (Sigma-Aldrich, Inc., St Louis, MO, USA) supplemented with protease inhibitors (Complete, Mini, EDTA-free Protease Inhibitor Cocktail, Hoffmann-La Roche, Basel, Switzerland). Samples were subsequently centrifuged at 10,000× *g* for 15 min at 4 °C. The supernatant was collected and stored at −80 °C until use. Protein concentration in tissue homogenates was determined by Bradford’s method (Quick StartTM Bradford Protein Assay, Bio-Rad Laboratories, Hercules, CA, USA).

### 2.5. ELISA Tests 

Blood and the aortic tissue homogenates were tested for MPO (Myeloperoxidase ELISA, DRG International, Springfield, NJ, USA), NGAL (Human NGAL ELISA Kit, BioPorto Diagnostics, Needham, MA, USA), neutrophil elastase (AssayMax Human Neutrophil elastase ELISA Kit, AssayPro St. Charles, MO, USA), IL-6 (IL-6 ELISA, DRG International, Inc. Springfield, NJ, USA), and TNF-a (AssayMax Human TNF-alpha ELISA Kit, AssayPro St. Charles, MO, USA) using commercially available enzyme-linked immunosorbent assays (ELISAs), according to the manufacturers’ instructions. The absorption was read at 450 nm using a plate reader (EnVision Multilabel Plate Reader; PerkinElmer, Waltham, MA, USA). Data standardisation in tissue biopsies used total protein concentration in the homogenates. The final concentrations of NGAL, MPO, and neutrophil elastase in the homogenates were expressed as ng/mg of total protein extract; TNF-a and IL-6 were given as pg/mg of total protein extract. Assays were performed twice with the four different tissue biopsies and serum or plasma in the same series to avoid bias due to assay variability. A 50-fold or 100-fold sample dilution was made if necessary using sample diluent.

### 2.6. Statistical Analyses

Data were analysed using commercial software (Statsoft Statistica 12; Tibco Software, Palo Alto, CA, USA). Descriptive statistics are given for all data. The normality assumption was examined using Shapiro–Wilk tests. The distributions of most sample parameters were not normal, and Levene tests showed heteroscedasticity in these cases. Therefore, nonparametric methods were used for all samples. Outlier measurements were performed. Extreme values, defined as exceeding above or below the median values by three times the interquartile range, were eliminated from the data sets. Spearman’s rank correlation coefficients were found for parameters from the samples from abdominal aortic aneurysms patients, separately for wall segments adjacent to thin intraluminal thrombus, adjacent to thick intraluminal thrombus, and in thin and thick intraluminal thrombus sites. Values of all five parameters were compared between the patient group and the control group using Mann–Whitney *U* tests. Wilcoxon signed-rank tests were used to compare values of analysed parameters in samples from patients taken at different AAA locations. Significance was defined at *p* < 0.05.

## 3. Results

### 3.1. Neutrophil Mediator Level Evaluation in Plasma/Serum

In Table 1, the demographic, clinical, and biochemical characteristics of patients with an AAA and control individuals are presented. The measured parameters, neutrophil elastase, MPO, and NGAL levels in blood samples were found to be significantly lower in AAA subjects than in the control group. The two groups did not differ in remaining blood parameters, including IL-6 and TNF-a.

### 3.2. Neutrophil Mediator Distribution in Abdominal Aortic Aneurysm

**Figure 2:**
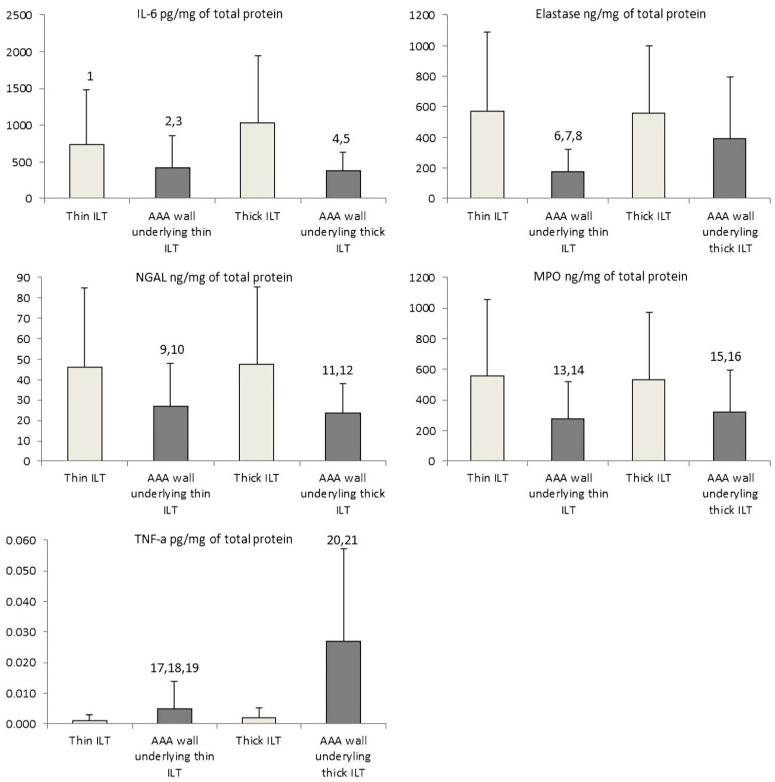
Wilcoxon matched pairs test of neutrophil mediators and inflammatory cytokines in parts of AAA. Bars represent standard deviation. Significant results: ^1^ thin ILT vs. thick ILT (*p* = 0.03), ^2^ wall adjacent to thin ILT vs. thin ILT (*p* = 0.03), ^3^ wall adjacent to thin ILT vs. thick ILT (*p* = 0.0004), ^4^ wall adjacent to thick ILT vs. thick ILT (*p* = 0.00002), ^5^ wall adjacent to thick ILT vs. thin ILT (*p* = 0.004), ^6^ wall adjacent to thin ILT vs. thin ILT (*p* = 0.01), ^7^ wall adjacent to thin ILT vs. thick ILT (*p* = 0.006), ^8^ wall adjacent to thin ILT vs. wall adjacent to thick ILT (*p* = 0.002), ^9^ wall adjacent to thin ILT vs. thin ILT (*p* = 0.006), ^10^ wall adjacent to thin ILT vs. thick ILT (*p* = 0.002), ^11^ wall adjacent to thick ILT vs. thick ILT (*p* = 0.0003), ^12^ wall adjacent to thick ILT vs. thin ILT (*p* = 0.0006), ^13^ wall adjacent to thin ILT vs. thin ILT (*p* = 0.0004), ^14^ wall adjacent to thin ILT vs. thick ILT (*p* = 0.0002), ^15^ wall adjacent to thick ILT vs. thick ILT (*p* = 0.003), ^16^ wall adjacent to thick ILT vs. thin ILT (*p* = 0.009), ^17^ wall adjacent to thin ILT vs. thin ILT (*p* = 0.02), ^18^ wall adjacent to thin ILT vs. thick ILT (*p* = 0.02), ^19^ wall adjacent to thin ILT vs. wall adjacent to thick ILT (*p* = 0.00005), ^20^ wall adjacent to thick ILT vs. thick ILT (*p* = 0.00005), ^21^ wall adjacent to thick ILT vs. thin ILT (*p* = 0.00004).

The results from measured AAA tissue parameters are shown graphically in Figure 2. As detected by ELISA, the IL-6, neutrophil elastase, NGAL, and MPO levels were higher in ILT segments than in AAA wall sections. In contrast, the levels of TNF-a were significantly higher in AAA wall segments than in ILT sections. Moreover, there were differences in measured parameter concentrations between thin and thick ILT sections. 

#### 3.2.1. Interleukin 6

Interleukin-6 levels were significantly higher in thick thrombus sections compared to thin ILT segments (1028 ± 921 vs. 742 ± 737, *p* = 0.03), wall adjacent to thin ILT (1028 ± 921 vs. 413 ± 438, *p* = 0.0004), and wall adjacent to thick ILT (1028 ± 921 vs. 379 ± 251, *p* = 0.00002). There were also significantly higher IL-6 concentrations in thin ILT than wall segments underlying thick ILT (742 ± 737 vs. 379 ± 251, *p* = 0.004) or adjacent to thin ILT (742 ± 737 vs. 413 ± 438, *p* = 0.03). 

#### 3.2.2. Neutrophil Elastase

Neutrophil elastase levels were increased in thin and thick ILT sections compared to the AAA wall sections underlying thin ILT (571 ± 519 vs. 176 ± 147, *p* = 0.01; 557 ± 443 vs. 176 ± 147, *p* = 0.006, respectively). Interestingly, neutrophil elastase levels were higher in wall segments underlying thick ILT than wall sections underlying thin ILT (394 ± 398 vs. 176 ± 147, *p* = 0.002). 

#### 3.2.3. Neutrophil Gelatinase-Associated Lipocalin

The ILT sections had significantly higher levels of NGAL compared with wall segments. NGAL concentrations were elevated in thick thrombus sections compared with walls adjacent to thick ILT (47 ± 38 vs. 23 ± 15, *p* = 0.0003) and walls adjacent to thin ILT (47 ± 38 vs. 27 ± 21, *p* = 0.002). Likewise, thin ILT had significantly higher levels of NGAL compared with parts of the wall adjacent to thick ILT (46 ± 39 vs. 23 ± 15, *p* = 0.0006) and wall underlying thin ILT (46 ± 39 vs. 27 ± 21, *p* = 0.006). There were no significant differences in NGAL levels between thick and thin segments of thrombus and AAA walls. 

#### 3.2.4. Myeloperoxidase

Myeloperoxidase content was higher in thin and thick ILT sections than the AAA wall section underlying thin ILT (560 ± 497 vs. 277 ± 241, *p* = 0.0004; 535 ± 440 vs. 277 ± 241, *p* = 0.0002, respectively) and thin and thick ILT sections compared to the AAA wall sections underlying thick ILT (560 ± 497 vs. 320 ± 274, *p* = 0.009; 535 ± 440 vs. 320 ± 274, *p* = 0.003, respectively). There were no significant differences in MPO levels between thick and thin segments of thrombus and AAA walls. 

#### 3.2.5. Tumour Necrosis Factor Alpha

The mean values of TNF-a concentrations were higher in both wall types compared to thrombus segments. Significantly more TNF-a was found in wall segments adjacent to thick thrombus than in any other AAA compartments (compared to AAA wall underlying thin ILT: 0.03 ± 0.03 vs. 0.005 ± 0.009, *p* = 0.00005; thin ILT: 0.03 ± 0.03 vs. 0.001 ± 0.002, *p* = 0.00004; thick ILT: 0.03 ± 0.03 vs. 0.001 ± 0.03, *p* = 0.00005). Similarly, TNF-a was significantly higher in the walls adjacent to thin ILT compared with the thick thrombus sections (0.005 ± 0.009 vs. 0.001 ± 0.003, *p* = 0.02) and thin thrombus sections (0.005 ± 0.009 vs. 0.001 ± 0.002, *p* = 0.02). There were no significant differences with TNF-a concentrations between thick and thin ILT.

### 3.3. Correlations between Neutrophil Mediator Levels within Thin and Thick ILT and AAA Wall Sections

Correlations between pairs of parameters measured at all AAA sections are shown in Table 2. The largest number of significant correlations were found within the AAA wall underlying thick ILT. In this region, NGAL concentrations correlated positively with both neutrophil elastase (r = 0.76, *p* = 0.0001) and MPO concentrations (r = 0.71, *p* = 0.0001); neutrophil elastase correlated positively with MPO concentrations (r = 0.84, *p* = 0.0001). Interestingly, similarly strong correlation patterns for NGAL and neutrophil elastase, and MPO were found in all AAA sections. Correlations with IL-6 were found mainly in thick AAA sites. This parameter correlated positively with NGAL and neutrophil elastase in AAA walls underlying thick ILT (r = 0.43, *p* = 0.011; r = 0.39, *p* = 0.017, respectively) and thick thrombus (r = 0.40, *p* = 0.018; r = 0.38, *p* = 0.024, respectively), as well as with MPO within AAA walls underlying thick ILT (r = 0.58, *p* = 0.0002). In addition, within AAA walls underlying thin ILT, IL-6 concentrations correlated positively only with MPO (r = 0.50, *p* = 0.002). Moreover, correlations between MPO and neutrophil elastase were found at all AAA sites (r = 0.87, *p* = 0.0001; r = 0.82, *p* = 0.0001; r = 0.85, *p* = 0.0001; r = 0.84, *p* = 0.0001, respectively, for thin and thick ILT sections, and AAA wall underlying thin and thick ILT). No significant correlations were found between TNF-a and other parameters in any group. 

### 3.4. Correlations of Neutrophil Mediator Levels between Thin and Thick ILT and AAA Wall Sections

Thick and thin intraluminal thrombus MPO and TNF-a levels correlated positively with their corresponding levels in adjacent walls (Table 3). Thick ILT neutrophil elastase and IL-6 levels correlated positively with their corresponding levels in the underlying AAA walls (r = 0.35, *p* = 0.035; r = 0.39, *p* = 0.022, respectively) but not in thin AAA regions. Hence, the correlation for NGAL was only observed between thin ILT and the underlying AAA wall (r = 0.33, *p* = 0.047). 

### 3.5. Correlations of Tissue and Plasma/Serum Neutrophil Mediator Levels with ILT Thickness and AAA Diameter

A correlation between measured parameters and ILT thickness was found only for IL-6 in thick thrombus sections (r = 0.36, *p* = 0.034). No statistically significant correlations were shown with AAA diameter. Likewise, no correlations were found between plasma/serum neutrophil marker levels in blood samples and thrombus thickness or AAA diameter. Interestingly, none of the studied neutrophil markers found in the thrombus and AAA walls showed a significant relationship to their blood concentrations using Spearman’s rank correlation analysis.

## 4. Discussion

Most studies thus far have reported the clinical and functional significance of ILT formation in AAA progression. Thrombus formation is achieved through various mechanisms, but a significant factor is disturbed blood flow. Altered hemodynamic conditions such as low wall shear stress, flow separation, and vortex ring formation were found in every stage of aneurysm development [40]. These conditions likely play a significant role in initiating and changing the pattern of ILT formation. The mechanical and physical features of this aneurysm component have been studied [4,5,6]. However, many authors have also noticed that an ILT is an essential source of proteases, inflammatory mediators, and cytokines [8,18,37,38]. Comparison of AAA regions with high ILT burden with low ILT burden might explain differences in inflammatory activities and clarify the inconsistent findings regarding the relationship between rupture location and ILT thickness.

In the present study, we evaluated the levels of several neutrophil mediators in aneurysmal tissues separated into thrombus and underlying wall material. We focused on comparing thin (<10 mm) and thick (>25 mm) sections of ILT and adjacent walls taken from one aneurysmal sac, as these regions seem to differ most in respect to physical and cellular composition. As expected, and in agreement with other studies, we showed increased levels of measured mediators in ILT compared with underlying AAA walls regardless of thickness, except with TNF-a, for which levels were statistically higher in the wall underlying thick ILT. Measured mediators are secreted by trapped neutrophils located in large quantities in the ILT and/or reach the thrombus and adjacent walls from the blood [8,18]. The presence of NGAL and other mediators probably reflects the activation of PMNs trapped inside ILT. Recent studies have shown that PMN mediators may result in continuous destruction of the thrombus, which facilitates its turnover and modulation of aortic wall destruction [8,25,41]. Thus, the presented results indicate that ILT might be the main source of neutrophil mediators in the AAA lesion.

Moreover, we found differences in measured marker concentrations between thin and thick AAA compartments, especially IL-6, TNF-a, and neutrophil elastase. Thick ILT showed higher levels of IL-6 than thin ILT and other AAA segments; AAA wall underlying thick ILT showed higher TNF-a levels than the wall underlying thin and other AAA segments, and AAA wall underlying thick ILT showed higher levels of neutrophil elastase than thin. These findings have been indirectly confirmed by other investigators who reported increased inflammatory infiltration in AAA wall adjacent to an ILT. Earlier studies showed that the microstructure of an aneurysm wall is significantly damaged and that neutrophils are also observed in the aneurysmal adventitia [21,42,43]. These infiltrating cells are probably recruited by massive elastin fragmentation and may further secrete inflammatory mediators and thus amplify extracellular matrix degradation [44]. Moreover, PMNs may be an important source of oxidative stress. Huang et al. suggested that a combination of high stress and depressed aortic walls in the presence of thick ILT might cause progression of inflammation in AAA walls [45]. Several other studies have shown that thicker ILT generates more hypoxia in the underlying aortic walls, which might induce an oxidant/antioxidant imbalance and increase oxidative stress levels, thereby enhancing vascular smooth muscle cell (VSMC) apoptosis and damage elastin fibres [46,47]. Wiernicki et al. showed that oxidative stress and proteolytic enzyme expression were enhanced in the outer media of walls covered by a thick thrombus [48]. In addition, as a result of hypoxia, the compensatory processes of angiogenesis intensify, and newly formed blood vessels are an additional pathway for the influx of pro-inflammatory cells into the AAA wall [49]. Thus, high concentrations of some PMN mediators in thicker AAA compartments may result from these processes. In a few studies, the release of inflammatory mediators was demonstrated to be significantly higher from the luminal layer of ILT than from the layers more toward the aneurysm wall [8,18,37,41]. However, it was not in all cases that a separation of thrombus and wall material was performed, nor were measurements compared of neutrophil mediators from different layers of the same thrombus. Moreover, dividing the ILT into three layers is only possible if the ILT is thick enough [18,37,41,50]. 

It has been suggested that the severity of the proteolytic and inflammatory processes might be proportional to the thickness of an ILT and that penetration of blood cells into aneurysm tissues may only occur up to 10 mm into the thickness of an ILT, possibly indicating a reduction in their passage into ILT thicker regions [51]. This would imply that a segment of thin ILT might be more permeable. From thick ILT, mediators and cells may reach the underlying aortic wall in relatively low concentrations. The result of this hypothesis would be an increased local release of proteolytic enzymes from thin AAA segments together with the altered structure of the underlying artery wall and a vicious circle of inflammation, increasing the risk of aneurysm rupture [52]. When this hypothesis is considered, the findings in the present study of strong positive correlations of measured mediators between thick ILT and adjacent wall sections as reported for all determined parameters (except NGAL) are surprising. These significant positive correlations suggest that PMN mediators probably do infiltrate thick AAA segments as well as thin ones. It should be mentioned that the mediators from plasma and those produced by ILT-trapped neutrophils were observed both in the thrombus and in adjacent aneurysm walls. Additionally, one study has shown that canaliculi, through which cells and proteins travel, are also present in abluminal layers of ILT and the AAA walls and are even larger than those in luminal layers, which could allow both larger molecules and cells to reach the wall [53,54]. The presence of such a leaky ILT and a spongy vessel wall that can allow proteases and other unfavourable molecules to accumulate and degrade components of the wall could ultimately lead to AAA diameter widening and subsequent rupture. ILT deposition is a dynamic process, building increasing ILT at varying AAA regions over time. AAAs with relatively more significant amounts of ILT may reflect a slower aneurysm growth with longer deposition times [55]. It should also be borne in mind that any AAA wall adjacent to thick thrombus most likely originally adjoined thin ILT, and further thrombus build-up may have covered the original luminal layer of the ILT. This process could trigger an evolution of properties of the ILT and underlying aortic wall. The thrombotic material’s mechanical properties may change significantly with the thrombus age, and, remarkably, older thrombi are related to aneurysm wall weakening [56]. As a result, thick ILT could contribute to the often chronic inflammation of the underlying aortic wall, with the sustained renewal of cellular activity at the lumen boundary through aggregation, entrapment, and recruitment of activated platelets and inflammatory cells. Consequently, the ILT could serve as a reservoir of a myriad of mediators that can be released and activated. One possible explanation is that, on the one hand, thinner ILT has a higher proteolytic activity, which could lead to a local weakening of the aneurysm wall [17,18,57,58]. Conversely, and perhaps later, it is believed that a thicker ILT enhances the inflammatory activity of the AAA wall by inducing local hypoxia of the AAA wall [46]. Processes under the influence of mediators released from neutrophils might weaken the structural integrity of an AAA vessel wall covered with thick thrombus. Further, proteolytic enzymes in the luminal layer of an ILT may aggravate this situation by causing fissures in the ILT, increasing the mechanical stress in the underlying wall, which could possibly contribute to a weakened AAA vessel wall. Recently, Ducas et al. demonstrated that AAA regions with thick ILT were associated with significant increases in inflammation compared with regions with thin ILT or with control tissue. In addition, thick ILT was associated with local AAA wall degeneration, as evidenced by increased elastin and collagen loss [39].

Several studies have implied that ILT thickness and AAA diameter influence AAA wall stability and contribute to aneurysm growth and rupture. In the present study, no correlations between tissue levels of neutrophil markers with either maximal thrombus thickness or maximal aortic diameter were observed, except for a positive correlation between IL-6 levels in thick ILT and the thickness of the ILT. These observations might suggest that IL-6 could promote thrombus formation through increased coagulation activity [59]. This is in agreement with previously published results by Houard et al., who reported no correlations between tissue MPO and MMP9/NGAL and maximal ILT thickness [37]. 

A general absence of correlations was also observed for blood neutrophil markers, suggesting that these markers are not directly linked to AAA morphology and might not be uniquely related to ILT and AAA wall activity. Interestingly, none of the studied mediators found in the aneurysmal tissues showed significant relationships to their levels in blood using Spearman’s rank correlation analysis, which further emphasises that the local thrombus microenvironment may be more important for its mediator content than the composition of neutrophil mediators in the peripheral blood. 

Concerning the neutrophil-derived markers, similar distribution patterns for NGAL, neutrophil elastase, and MPO were observed in the present study. Notably, significant correlations were observed between NGAL and neutrophil elastase and MPO within thin and thick ILT regions. Interestingly, similar correlation patterns were observed for NGAL, MPO, and neutrophil elastase in AAA wall underlying thin and thick ILT (Table 2). Correlations between MPO and both NGAL and neutrophil elastase concentrations (markers of neutrophil degranulation) may suggest that neutrophils could be the main source of these inflammatory biomarkers within the ILT and AAA wall. These results also suggest a potential activated state of neutrophils in AAA tissues. The expression of NGAL can be induced by several cytokines and growth factors, including MPO and TNF-a [22]. Some of these markers activate and provoke the chemotaxis of neutrophils, which are the main source of NGAL and other mediators [18,19]. This indicates that neutrophils that may produce neutrophil chemotactic factors might amplify their own recruitment. Damage caused to the endothelium contributes to the formation of a vicious cycle of inflammation by intensifying the chemotaxis of pro-inflammatory cells. 

Surprisingly, in the present study, the plasma concentrations of neutrophil elastase, MPO, and NGAL were elevated in the control group versus AAA patients. In the case of the remaining markers, we did not observe statistically significant differences between the AAA patients and the control group. Some substances which originate from activated neutrophils in AAA tissue can be found at increased levels in the peripheral blood, as already described for neutrophil elastase, NAGL, inflammatory cytokines, and others [35,60]. It should also be taken into account that particular drugs may influence cytokine levels, and treatments could influence oxidative stress and inflammatory status. Patients and controls showed similar risk factors and medications, suggesting that the differences in plasma of measured parameters could be associated with the disease progression or might be related to the relatively small sample size.

Certain limitations in the present study need to be addressed: (1) The low number of patients with AAA enrolled in the presented study collected at one institute. It should be noticed that there are a decreasing number of open surgeries and an increasing number of endovascular AAA repairs, which make it difficult to collect a desired number of patients. (2) We measured the immune reactivity of NGAL, neutrophil elastase, and MPO in the plasma and tissue of patients with aneurysmal disease. These assays do not show the enzymatic activity of the measured mediators. (3) The aneurysmal tissues were obtained from surgery patients in which the AAA had obtained a particular size, and therefore an earlier stage of aneurysm may show different alterations than described here. (4) Heterogeneity in inflammatory processes in the AAA wall might have biased the results.

## 5. Conclusions

In conclusion, the present study showed that levels of neutrophil elastase and TNF-a were significantly higher in aneurysmal walls covered by thick ILT (>25 mm) and that concentrations of IL-6 in thick ILT were higher than in thin AAA layers. In addition, significant positive correlations were observed for neutrophil mediators between thick ILT layers and adjacent walls and between thin sections, suggesting that these mediators probably infiltrate thick AAA compartments as well as thin. These observations might support that neutrophil mediators and inflammatory cytokines differentially accumulate in AAA tissues according to ILT thickness. Moreover, the increased level of selected neutrophil mediators might support the existence of a proinflammatory state in thicker AAA segments (compared with thinner AAA sections), which probably preferentially weaken the AAA wall in this region. Therefore, studies investigating therapies to reduce the ILT thickness may be warranted to evaluate their effect on AAA growth and rupture. A larger study is, however, needed to confirm these observations.

## Figures and Tables

**Table 1:** Demographic, clinical, and biochemical characteristics in subjects with an abdominal aortic aneurysm (AAA) and control group.

		AAA (*n* = 36)	Control (*n* = 29)	*p*
Age (years)		71 ± 8	63 ± 7	0.00009
Sex (male/female)		27 (75)/9 (25)	21 (69)/9 (31)	0.589
Current smoker		17 (47)	18 (62)	0.232
Diabetes mellitus		7 (19)	7 (24)	0.647
Hypertension		23 (64)	17 (59)	0.664
CVD	Myocardial infarction	2 (6)	6 (21)	0.065
	Stroke	2 (6)	5 (17)	0.131
DVT		1 (3)	1 (3)	0.876
Statin therapy		3 (8)	8 (28)	0.039
MPO (ng/mL) ^‡^		491 ± 183	601 ± 204	0.025
NGAL (ng/mL)		190 (129–351)	439 (242–733)	0.001
Elastase (ng/mL)		906 (535–1317)	1237 (842–1865)	0.045
IL-6 (pg/mL)		40.0 (21.6–79.4)	32.8 (16.8–58.3)	0.229
TNF-a (pg/mL)		0.001 (0.001–0.008)	0.001 (0.001–0.004)	0.443
AAA diameter ^‡^		59 ± 12		
ILT thickness ^‡^		32 ± 10		

Unless indicated otherwise, values are data reported as arithmetic mean and 95% confidence intervals in parentheses or counts with percentages (categorical variables); ^‡^ mean ± standard deviation (continuous variables); CVD denotes cardiovascular disease; DVT—deep vein thrombosis; MPO—myeloperoxidase; NGAL—neutrophil gelatinase-associated lipocalin; IL-6—interleukin-6; TNF-a—tumour necrosis factor alpha.

**Table 2:** Correlations between pairs of parameters measured at thin and thick regions of intraluminal thrombus (ILT) and adjacent walls of abdominal aortic aneurysms (AAA).

Correlation	AAA Wall Parts Adjacent to ILT				Intraluminal Thrombus			
	Site Adjacent to Thin ILT		Site Adjacent to Thick ILT		Site Thin		Site Thick	
	R	*p* Corrected *p*-Values	R	*p* Corrected *p*-Values	R	*p*-Values	R	*p* Corrected *p*-Values
NGAL vs. elastase	0.81	0.0001	0.76	0.0001	0.85	0.0001	0.78	0.0001
NGAL vs. MPO	0.75	0.0001	0.71	0.0001	0.90	0.0001	0.77	0.0001
NGAL vs. IL-6	0.30	0.079	0.43	0.011	0.23	0.184	0.40	0.018
NGAL vs. TNF-α	−0.02	0.920	−0.25	0.162	0.08	0.637	−0.06	0.754
Elastase vs. MPO	0.85	0.0001	0.84	0.0001	0.87	0.0001	0.82	0.0001
Elastase vs. IL-6	0.28	0.122	0.39	0.017	0.19	0.276	0.38	0.024
Elastase vs. TNF-α	−0.11	0.554	−0.19	0.252	0.27	0.222	0.17	0.341
MPO vs. IL-6	0.50	0.002	0.58	0.0002	0.20	0.242	0.23	0.174
MPO vs. TNF-α	−0.01	0.989	−0.11	0.505	0.15	0.421	0.15	0.400
IL-6 vs. TNF-α	0.27	0.121	0.23	0.170	0.32	0.066	0.16	0.388

R—Spearman correlation coefficient; NGAL—neutrophil gelatinase-associated lipocalin; MPO—myeloperoxidase; IL-6—interleukin 6; TNF-a—tumour necrosis factor alpha. Units are given in Figure 2. Underlined values show significant correlations.

**Table 3:** Correlations of inflammatory markers or cytokines between parts of intraluminal thrombus (ILT) or parts of adjacent walls of abdominal aortic aneurysms (AAA).

Correlation	Thin Parts		Thick Part	
	Between Thin ILT and Adjacent Walls of AAA		Between Thick ILT and Adjacent Walls of AAA	
	R *p* Corrected *p*-Values	*p* *p* Corrected *p*-Values	R *p* Values	*p* *p* Corrected *p*-Values
NGAL	0.33	0.047	0.28	0.111
Elastase	0.31	0.090	0.35	0.035
MPO	0.35	0.034	0.52	0.001
IL-6	0.18	0.040	0.39	0.022
TNF-a	0.39	0.028	0.68	0.0001

R—Spearman correlation coefficient; NGAL—neutrophil gelatinase-associated lipocalin; MPO—myeloperoxidase; IL-6—interleukin 6; TNF-a—tumour necrosis factor alpha. Units are given in Figure 2. Underlined values show significant correlations.

## Data Availability

Data are available on request due to restrictions, e.g., privacy or ethical. The data presented in this study are available on request from the corresponding author. The data are not publicly available due to reasons of sensitivity, e.g., human data.
